# Factors associated with urinary tract infections among HIV-1 infected patients

**DOI:** 10.1371/journal.pone.0190564

**Published:** 2018-01-11

**Authors:** Agata Skrzat-Klapaczyńska, Bartłomiej Matłosz, Agnieszka Bednarska, Marcin Paciorek, Ewa Firląg-Burkacka, Andrzej Horban, Justyna D. Kowalska

**Affiliations:** 1 Medical University of Warsaw, Department for Adult's Infectious Diseases, Warsaw, Poland; 2 Hospital for Infectious Diseases, HIV Out-Patient Clinic, Warsaw, Poland; Benue State University College of Health Science, NIGERIA

## Abstract

**Background:**

Urinary tract infections remain an important yet underinvestigated clinical problem among HIV infected patients. Here we analyze factors associated with its occurrence and the spectrum of bacterial pathogens identified in the group of patients followed at the HIV Out-Patient Clinic in Warsaw.

**Methods:**

Clinic database collected all medical information on patients routinely followed since 1994 to 2015. All patients with available urine culture were included into analyses, only the first culture was included. In statistical analyses logistic regression models were used to identify factors associated with positive culture.

**Results:**

In total 608 patients had urine culture performed, 176 (28.9%) were females and 432 (71,1%) were males, 378 (62.2%) registered in care before/in 2007, 258 (42.4%) infected through homosexual contact. Median baseline lymphocyte CD4+ count was 385 (IQR:204–565) cells/μl and median nadir lymphocyte CD4+ count 197 (86–306) cells/μl. One hundred and eighteen patients were actively infected with HCV, as defined by positive real-time PCR. In total 141 (23.2%) patients had positive urine culture, the most common bacterial pathogen was *E*.*coli* (58.2%) and *E*. *faecalis* (12.8%). Patients with urinary tract infection were more likely to be female (51.8% vs. 22.1%, p<0.0001), infected through other than homosexual mode (80.1% vs. 50.7%, p<0.0001), with lower nadir CD4 count (139 vs. 221 cells/μl, p<0.0001) and lower baseline HIV RNA (4.02 vs. 4.35 log copies/ml, p = 0.01) and less likely to be HCV RNA positive (26.9% vs. 49.2%, p = 0.01). In multivariate regression model being registered before/in 2007 (OR = 2.10; [95%CI: 1.24–3.56]), infected through other than homosexual mode (2.05;[1.18–3.56]) and female gender (2.14;[1.33–3.44]) were increasing and higher nadir CD4+ count decreasing (0.92;[0.85–0.99]) the odds of urinary tract infection.

**Conclusions:**

We have identified that almost one third of patients had urinary tract infections with non-typical bacterial pathogens. Population with increased odds of urinary tract infections are women, patients infected through other than homosexual contacts and those registered before 2007.

## Introduction

Urinary tract infection is the presence of pathogenic microorganisms in specified amount. Positive urine culture is defined when it shows a bacterial colony count of greater than or equal to 10^3^ colony-forming units per μl of a typical urinary tract organism [1]. They are the most frequent community-acquired infections in the world and the most common pathogens are *E*. *coli* [1, 2].

Urinary tract infections, both symptomatic and asymptomatic, are serious public healthcare problems decreasing the quality of life and leading to work absence [3].

Specific groups of people are at increased risk of urinary tract infection. Vulnerable populations are women, especially during pregnancy, infants and elderly patients [1, 4–6]. Also certain conditions may increase susceptibility to infections i.e. spinal cord injuries, urinary catheters, diabetes, multiple sclerosis, immunodeficiency and underlying urologic abnormalities [7, 8]. HIV positive patients are also prone to urinary tract infections. The incidence of urinary tract infections in HIV population is clearly related to infection and immune function, determined by lymphocytes CD4+ cells count [9, 10]. As confirmed by observational studies the incidence of various bacterial infections in HIV-infected patients, including urinary tract infections, is inversely correlated with lymphocyte CD4+ count [11]. It is therefore interesting to note that although the wide introduction of antiretroviral therapy has dramatically reduced morbidity related to AIDS, non-AIDS defining infections remain an important and frequent clinical problem[10]. This may result from increased frequency of non-HIV related diseases in the HIV population, such as diabetes and glucose metabolism disturbance, liver cirrhosis and metabolic syndrome [12–16].

Moreover the HIV population in Poland is aging, bringing new risk factor for non-HIV related infections. It is therefore crucial to observe the burden of such infections, its outcomes and change in clinical characteristics [17].

It is also noted that the spectrum of pathogens is more broad and diverse than in general population including less common microorganisms [18].

Improving knowledge on the prevalence and prognostic factors for urinary infections in HIV patients along with better recognition of the causative pathogens could substantially improve current diagnostic and treatment guidelines translating into better prognosis for HIV- positive patients [19].

Therefore the objectives of this study were to analyze factors associated with its occurrence and the spectrum of bacterial pathogen identified in the group of patients followed at the HIV Out-Patient Clinic in Warsaw.

## Methods

Clinic database of the HIV Out-Patient Clinic in Warsaw collects all medical information on patients routinely followed since 1994 to 2015. For this set of analyses all patients with available urine culture were included and only the first urine culture was considered.

Urinary tract infection is defined as the presence of microbial pathogens in specified amount. Positive urine culture is defined when it shows a bacterial colony count of greater than or equal to 10^3^ colony-forming units per μl of a typical urinary tract organism (1). For the identification of pathogens in urine Hoeprich method was used, wherein the urine is plated on the MacConkey and CLED substrate. For HIV RNA identification Abbott RealTime HIV-1 Test was used. To mark CD4/CD8, Blood samples were collected by venipuncture into a sterile BD Vacutainer® EDTA (ethylenediaminetetraacetic acid) blood collection tube. The identification, determination of the percentages and absolute counts of mature human T lymphocytes (CD3+), helper/inducer (CD3+CD4+) T lymphocytes and suppressor/cytotoxic (CD3+CD8+) T lymphocytes in erythrocyte-lysed whole blood was performed with use of the commercially available single tube—BD Tritest—three-color direct immunofluorescence reagent. The antybodies were stained with: CD4 fluorescein isothiocyanate (FITC)/CD8 phycoerythrin (PE)/CD3 peridinin chlorophyll protein (PerCP). The quantitation of HCV RNA in plasma was performed with using two commercially available HCV real time PCR assays: the Abbott m2000 sp/rt RealTi*m*e HCV assay (Abbott Labs Illinois, USA) or the Roche COBAS Ampliprep/COBAS Taqman HCV Quantitative Test v2.0 (Roche Molecular Systems, Pleasanton CA, USA). Both tests are *in vitro* reverse transcription-polymerase chain reaction nucleic acid amplification assays.

In statistical analyses non-parametric tests were used for the group comparison as appropriate. Logistic regression models were used to identify factors associated with positive urine culture. Variables tested in univariate models with p<0.1 were included into multivariate models.

All tests of significance were two-sided and a confidence interval of 95% was accepted.

All analyses were performed using Statistical Analysis Software Version 9.3 (Statistical Analysis Software).

### Ethical approvals

The study has been approved by the Bioethical Committee of the Medical University of Warsaw (Nr AUBE/102/15).

The participants agreed for diagnosis and treatment in HIV-Out Patient Clinic in Warsaw, but they didn't provide their written or verbal informed consent to participate in this study. Clinic database collects all medical information on patients routinely followed since 1994. The IRB that approved the study also approved the fact that consent was not needed.

The information collected were anonymized upon data export performed by authorised personell not linked to the study team. Patients' personal identification was not possible at any stage of the project and physicians involved both in the study and patients' care were fully blinded.

## Results

**Fig 1:**
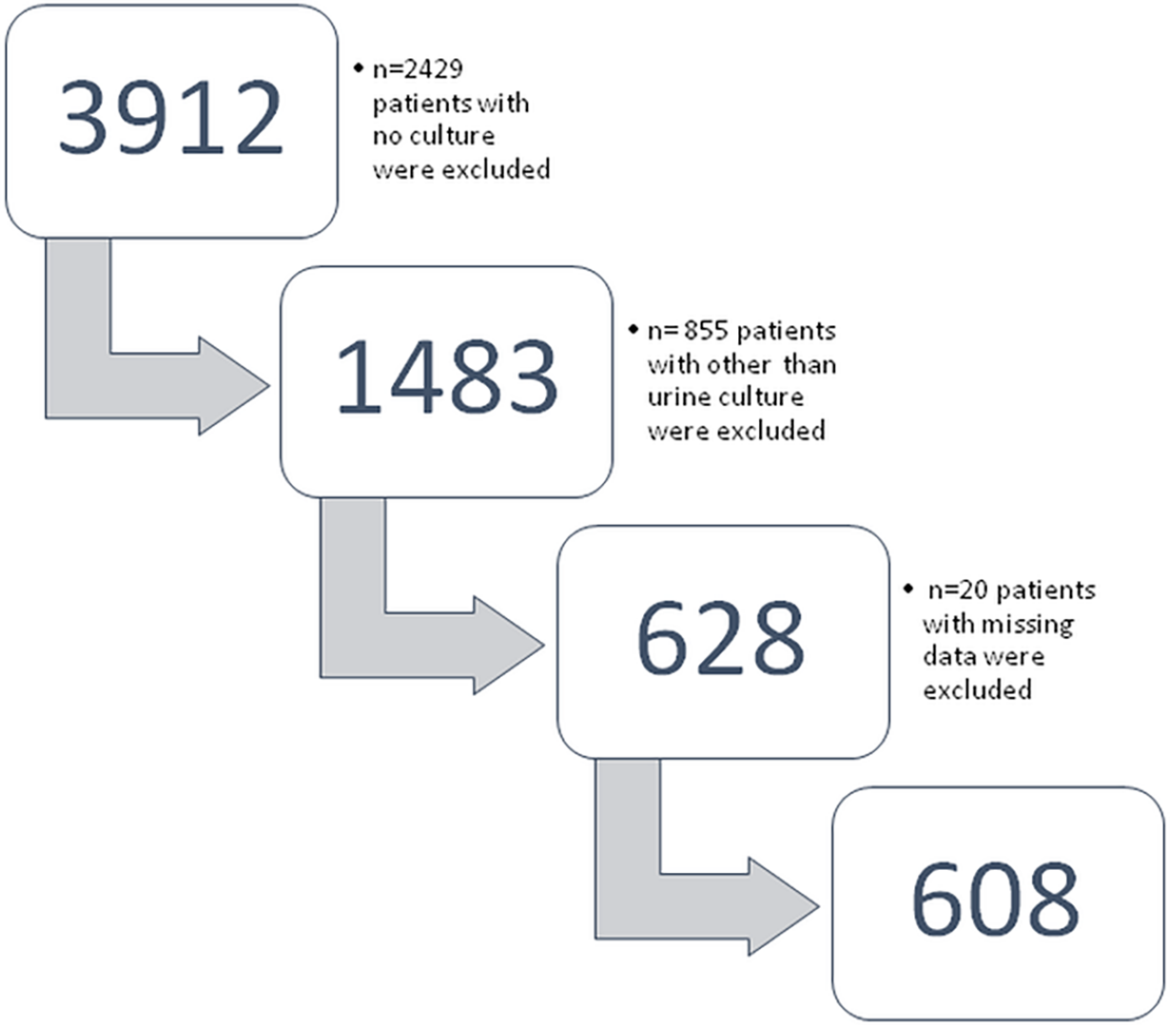
Patients' inclusion into analyses according to STROBE diagram.

In the electronic database 3912 patients had urine culture performed. During the verification of the study population, 2429 patients with no culture were excluded. In addition, 855 patients with other than urine culture were excluded. Moreover, 20 patients with missing data were excluded from the study (Fig 1).

Finally, 608 patients, who met the criteria of the study, took part in the analysis.

In total 608 patients had urine culture performed, 176 (28.9%) were females and 432 (71,1%) were males, 378 (62.2%) registered in care before/in 2007, 258 (42.4%) infected through homosexual contact. Median baseline lymphocyte CD4+ count was 385 (IQR:204–565) cells/μl and median nadir lymphocyte CD4+ count was 197 (86–306) cells/μl. One hundred and eighteen patients (19.4%) were actively infected with HCV. Five hundred eighty eight (96.7%) patients were on antiretroviral treatment, of them 420 (73.0%) on protease inhibitor based regimen and 147 (25.2%) on non-nucleoside reverse transcriptase inhibitor based regimen.

One hundred and forty one (23.2%) patients had positive urine culture, the most common pathogens were *E*. *coli* (58.2%) and *E*. *faecalis* (12.8%),Table 1.

**Table 1:** Bacterial pathogens identified by urine culture.

Pathogens identified by urine culture:	N	%
**Total number**	**141**	**100**
*Escherichia coli*	82	58.2
*Enterococcus faecalis*	18	12.8
*Staphylococcus epidermidis*	7	5.0
*Streptococcus agalactiae*	4	2.8
*Pseudomonas aeruginosa*	4	2.8
Mixed infection with two different patogens	4	2.8
*Proteus mirabilis*	3	2.1
*Streptococcus spp*	3	2.1
*Staphylococcus saprophyticus*	2	1.4
*Klebsiellapneumoniae*	2	1.4
**Pathogens with <1% prevalence:**		
*Acinetobacterbaumanii*	1	0.7
*Alcaligenes species*	1	0.7
*Candida albicans*	1	0.7
*Enterobactercloace*	1	0.7
*Klebsiellaoxytoca*	1	0.7
*Morganellamorganii*	1	0.7
*Pseudomonas spp*	1	0.7
*Serratiaodorifera*	1	0.7
*Staphylococcus aureus*	1	0.7
*Staphylococcus spp*	1	0.7
*Stenotrophomonasmaltophilia*	1	0.7
*Trichomonasvaginalis*	1	0.7

Patients with urinary tract infection were more likely to be female (51.8% vs. 22.1%, p<0.0001), infected through other than homosexual mode (80.1% vs. 50.7%,p<0.0001), with lower nadir lymphocyte CD4+ count (139 vs. 221 cells/μl, p<0.0001) and lower baseline HIV RNA level (4.02 vs. 4.35 log copies/ml, p = 0.01). They were also less likely to be HCV RNA positive (26.9% vs. 49.2%, p = 0.01) and had comparable HCV RNA levels (6.01 vs. 5.99 log copies/ml, p = 0.47). The rate of urinary infections was greater in patients being registered before/in 2007 (82.3% vs. 56.1% p<0,0001)Table 2. There was no difference between the groups in terms of proportion of patients on antiretroviral therapy (96.4 vs. 96.8, p = 0.79).

**Table 2:** Baseline characteristics for the groups of patients with positive and negative urine culture.

Characteristic Total N = 608	N total	Positive urine culture N = 141	Negative urine culture N = 467	P value
	N(%)			
Female gender	176 (28.9)	73 (51.8)	103 (22.1)	<0.0001
In care before/in 2007	378 (62.2)	116 (82.3)	262 (56.1)	<0.0001
Risk group				
Heterosexual contacts	122 (20.1)	41 (29.1)	81 (17.3)	<0.0001
Homosexual contacts	258 (42.2)	28 (19.9)	230 (49.2)	
Injecting drug use	162 (26.6)	52 (36.9)	110 (23.5)	
Other	18 (2.96)	9 (6.4)	9 (1.9)	
Unknown	48 (7.9)	11 (7.8)	37 (7.9)	
Positive HCV antibody	169 (29.4)	65 (48.1)	104 (23.7)	<0.0001
Positive HCV RNA	118 (19.4)	38 (26.9)	80 (17.1)	0.01
Baseline lymphocyte CD4+ count <350 cells/μl	270 (44.4)	71 (50.4)	199 (42.6)	0.10
Antiretroviral therapy—yes/no	588(96.7)/ 20(3.3)	136(96,45)/ 5(3.5)	452(96.8)/ 15(3.2)	0.79
Fusion inhibitors	7(1.2)	1(0.7)	6(1.3)	0.68
Integrase inhibitors	9(1.5)	1(0.7)	8(1.8)	
Non-Nucleoside reverse transcriptase inhibitors	147(25.2)	32(23.5)	115(25.7)	
Protease inhibitors	420(72.0)	102(75.0)	318(71.1)	
	Median (IQR)			
Age at registration in years	31.6 (26.6–39.2)	30.7 (24.9–39.3)	31.9 (27.0–38.0)	0.27
Nadir lymphocyte CD4+ count in cells/ μl	197 (86–306)	139 (60–225)	221 (107–322)	<0.0001
Baseline lymphocyte CD4+ count in cells/ μl	385 (204–565)	344 (160–562)	393 (228–570)	0.13
Baseline HIV RNA log copiel/ml	4,26 (3,36–4,93)	4,02(3,06–4,76)	4,35(3,49–4,00)	0.01
Highest proteinuria n = 190	0.51 (0.28–1.26)	0.93 (0.43–2.40)	0.45 (0.25–0.96)	0.002
HCV RNA n = 118 log copies/ml	5,99(5,20–6,49)	6,01(5,06–6,39)	5,99(5,28–6,50)	0.47

In univariate regression models being registered before/in 2007 (OR = 3.63; [95%CI:2.27–5.80]), not being infected through homosexual mode (3.92;[2.49–6.15]), female gender (3.79;[2.55–5.64]), positive HCV RNA (1.78; [1.15–2.78]) were increasing and higher nadir lymphocyte CD4+ count (0.85;[0.80–0.92]), model being on ARV (0.90; [0.322–2.529]) was decreasing the odds of first urinary tract infection. After adjustment for variables tested as significant in multivariate models, being registered before/in 2007 (OR = 2.10 [95%Cl:1.24–3.56]), not being infected through homosexual mode (2.05 [1.18–3.56]), female gender (2.14 [1.33–3.44]) were increasing and higher nadir lymphocyte CD4+ count (0.92 [0.85–0.99]) decreasing the odds of first urinary tract infection. After adjustment positive HCV RNA (0.96 [0,59–1.56]) was no longer a significant factor for urinary tract infection in HIV-infected patients, Table 3.

**Table 3:** Univariate and multivariate logistic regression models for positive urine culture.

	Univariate		Multivariate*	
Variable	OR (95% CI)	P value	OR (95% CI)	P value
Age by 1 year older	0.99 (0.97–1.01)	0.38	-	-
Nadir CD4 by 50 cells/ul higher	0.85 (0.80–0.92)	<0.0001	0.92 (0.85–0.99)	0.03
Baseline CD4 by 50 cells/ul higher	1.01 (0.99–1.02)	0.54	-	-
Highest HIV RNA by 4 log higher	1.00 (0.99–1.00)	0.87	-	-
Highest proteinuria by 0.5 mg higher	1.01 (0.99–1.02)	0.32	-	-
Antiretroviral therapy yes/no	0.90 (0.322–2.529)	0.84	-	-
Registered before/in 2007	3.63 (2.27–5.80)	<0.0001	2.10 (1.24–3.56)	0.006
Female gender	3.79 (2.55–5.64)	<0.0001	2.14 (1.33–3.44)	0.001
Other than homosexual mode of HIV infection	3.92 (2.49–6.15)	<0.0001	2.05 (1.18–3.56)	0.01
HCV RNA positive	1.78 (1.15–2.78)	<0.0001	0.96 (0.59–1.56)	0.86

* Adjusted for all variables significant (p<0.1) in univariable analyses

## Discussion

In our study one in four of observed patients had positive urine culture and the most common pathogen was *E*.*coli* (58.2%), followed by *E*.*faecalis* (12.8%). We have identified that almost one third of patients had urinary tract infections with non-typical pathogens.

In the study by Schonwald et al. among 96 HIV-positive patients the prevalence rate of urinary tract infections among HIV/AIDS patients in Zagreb was high (41%). *Enterococci* were the most frequent isolates in this study, whereas *Escherichia coli* was most frequently isolated in 314 non HIV-infected controls [9].

Comparably to our findings in Indian study of 350 HIV-positive patients, symptomatic urinary tract infection was observed in 24.3% of cases and *E*. *coli* was the most common aetiological agent of urinary tract infection. The other isolates were *Staphylococcus aureus* (5%), *Klebsiella pneumoniae* (4%), *Enterococcus faecalis* (2%), *Pseudomonas aeruginosa* (1%), *Proteus spp*. (1%) and *Staphylococcus epidermidis* (1%) [20].

In contrast to our work and the above mentioned studies a South-Western Nigerian study showed that in asymptomatic bacteriuria in HIV-positive patients *Klebiella spp*. was slightly higher in prevalence than *E*. *coli*. This is a big difference compared to other studies, where *E*. *coli*, both in HIV-positive and HIV-negative individuals is the dominating microorganism. Similar results were also reported by Gugino et al.[21].The most likely cause is that HIV-positive patients are at higher risk for infections due to hospital associated pathogens such as *Klebsiella spp*. This is due to more frequent necessity of hospitalization for patients with immunodeficiency and thus more frequent infections requiring hospitalization.

In our study we have registered outpatients and so they may have rather community-acquired pathogens and that is why *E*.*coli* was the most common isolated pathogen. Also in general population urinary infections are mostly caused by *E*. *coli* (80% to 85% of cases) [1]. This is well in line with our patient population being in majority on antiretroviral therapy, thus at lesser risk of both HIV and non-HIV infections, as well as with causative pathogens more comparable to those identified at non-HIV population.

Being registered before/in 2007, infected through other than homosexual mode and female gender in our study increased the odds of urinary tract infection, while higher nadir lymphocyte CD4+ count was decreasing the odds of first urinary tract infection.

Patients registered in/before 2007 in HIV care in Poland were likely to receive antiretroviral treatment with lymphocyte CD4+ count below 350 cells/μl. The regimens used at that time were also less likely to contain newer antiretroviral drugs such as tenofovir, integrase inhibitors and novel protease inhibitors or non-nucleoside reverse transcriptase inhibitors. Due to low statistical power we were not able to analyze separate drug effect, which could explain the doubled risk of urinary tract infection.

The prevalence in general population is higher in female and our study showed that 51.8% of studied women had positive urine culture as compared to only 22.1% in men. The female gender was increasing the odds of urinary infection by 114%. Similar results were obtained in the Indian study where 28% women and 22% of men presented with urinary tract infection [20].This is well in line with other studies and attributed to the shorter length of urethra in women and its proximity to the anus [22, 23].

It has been observed that those with low lymphocyte CD4+ counts (<200 cell/μl) are at significantly higher risk of asymptomatic bacteriuria and are therefore more likely to develop urinary tract infection [3].

De Pinho and colleagues investigated the relation between immune status and asymptomatic bacteriuria or urinary tract infection in HIV-positive men. They examined 415 patients showing observed that the risk of both asymptomatic and symptomatic urinary infection was increasing with decreasing immune function [24].

The percentage of HIV-positive patients with active HCV infection ranges from 10% to 50% worldwide [25–28]. In our study in total 118 (19.4%) patients were actively infected with HCV. Co-infection with HIV and HCV has a significant influence on the rapid progression of liver fibrosis compared with HCV monoinfected patients [29, 30].

HIV-infected patients who are also infected with HCV, as a result of accelerated liver fibrosis fast leading to cirrhosis of the liver, have an additional factor of immune deficiency [31, 32]. In our study in univariate regression analyses active HCV infection was increasing the odds of first urinary tract infection, however after adjustment for other cofounders HCV RNA was no longer a significant factor.

There are some limitations to our study which need to be mentioned. First off all data on clinical symptoms and indications for urine culture were not available for these analyses. This could result in underreporting of bacterial infections of the urinary tract. Secondly we did not investigate patients with the clinically based diagnosis and without urine culture performed. Therefore our findings can only be referred to population of HIV-positive patients who had urine culture based diagnosis.

We have done sensitivity analysis regarding long term of observational period for the whole cohort limiting the model to patients registered in care on/after 01Jan 2007 seen same trends yet due to lower number of patients the trends were no longer significant.

Although antiretroviral treatment significantly improved survival and diminished morbidity due to AIDS, this trend is not so evident in relation to non-AIDS conditions. Recent data from EuroSIDA study showed that the incidence of deaths due to bacterial infections has not changed significantly despite prolonging time of exposure to antiretroviral treatment [33]. This underlines the fact that bacterial infections are still valid and important events even in the era of effective therapy. As ongoing significant clinical problem they may significantly reduce the netto benefit received from the use of antiretrovirals treatment.

The review of existing literature shows that the knowledge about the nature of bacterial infections is still very limited, so the future direction of observational studies should focused on monitoring this important end-points as well as to investigate the range of causative pathogens. This is especially relevant with recent reports from the START study suggesting that therapy should be started as soon as possible and the impact of such intervention on the occurrence of such endpoints as infection is yet to be established [34].
